# Internet Use and Cognitive Functioning at the Interaction of Age and Gender Among Older Adults

**DOI:** 10.1093/geroni/igab046.2650

**Published:** 2021-12-17

**Authors:** Kun Wang, Zainab Suntai, Xiayu Chen

**Affiliations:** 1 University of Alabama, Tuscaloosa, Alabama, United States; 2 University of Illinois at Urbana-Champaign, New York, New York, United States

## Abstract

The positive relationship between internet use and cognition has long been documented in the gerontology literature, and researchers are consistently finding that internet use engages the brain in a way that improves cognitive functions such as multitasking, information processing and executive thinking. While there are numerous studies examining this association, few studies have explored the three-way interaction between age, gender, and internet use on cognition. This study aimed to examine the gendered moderation effect of age on the relationship between internet use and cognition among older adults. The study sample was derived from the 2016 Health and Retirement study, which is a biennial longitudinal panel study of adults aged 50 and older in the United States. Multilinear regression models were used to examine the three-way interaction of age, sex and internet use on cognition while controlling for other covariates. Results showed that women gained a greater increase in cognition as a result of internet use as they became older, while men had the same amount of increase in cognition as a result of internet use regardless of age. This indicates that internet use can be a positive agent in improving cognition among older adults regardless of age and sex, and interventions should focus on increasing internet use among older adults, to ensure equitable access to the benefits of internet use on cognition.

